# Innovations in bulk photovoltaics: design strategies for boosted photocurrent

**DOI:** 10.1038/s41377-025-01764-7

**Published:** 2025-02-20

**Authors:** Akhil Sreevalsan, Hyosung Choi

**Affiliations:** Department of Chemistry, Research Institute for Convergence of Basic Science and Research Institute for Natural Sciences, Hanyang University, Seoul, 04763 Republic of Korea

**Keywords:** Optical materials and structures, Photonic devices

## Abstract

The limitations imposed by low contact resistance, restricted polarization access, and tensile strain in bulk photovoltaic systems were mitigated by the engineering and optimization of edge semimetal contacts using Bi/Au. Improved bulk PV photocurrent and intriguing prospective applications are made possible by this effort.

## Graphical abstract

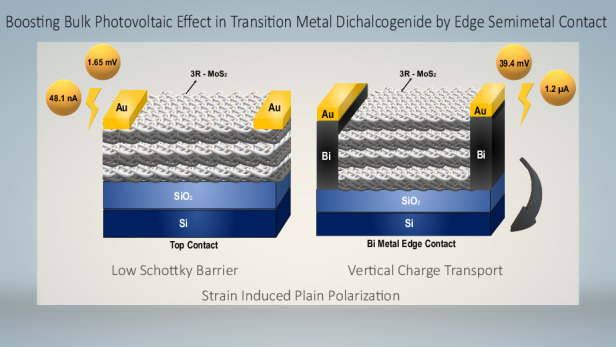

Over the past few decades, advancements in photovoltaic technologies have primarily centered around the conventional p-n junction-based photovoltaic device, which relies on an intrinsic electric field for charge separation^1^. However, the bulk photovoltaic effect (BPVE) offers a new approach, generating photocurrent in semiconductors under uniform illumination due to the asymmetric distribution of photo-excited carriers in momentum or real space^2^. The intrinsic homogeneity of BPVE systems, along with their potential to exceed the Shockley–Queisser efficiency limit, makes BPVE a promising candidate for next-generation photovoltaic technologies^3^. Traditional non-centrosymmetric materials utilized in BPVE systems have an array of challenges despite their promise. These drawbacks include restricted spectrum response and larger bandgaps, which lead to lower photocurrent generation, posing a challenge for maximizing the efficiency of these materials in photovoltaic applications. The advent of transition metal dichalcogenides (TMDs) (3R-MoS_2_) has addressed these limitations, showcasing superior light-matter interaction and high carrier mobility that enhance both light absorption and charge transport^4,5^. Additionally, their layered structure enables versatile engineering and strain modulation, optimizing the dynamics of photo-generated carriers for efficient photovoltaic operation^6^.

The research by Yoshihiro Iwasa and a group from the University of Tokyo, on strain engineering was a significant leap in the field of BPV materials^5^. This breakthrough has expanded the potential of BPV materials, particularly with 3R-MoS_2_, opening new avenues for exploration and development in the field. Strain-induced polarization has been found to significantly enhance the bulk photovoltaic effect (BPVE) in 3R-MoS_2_, leading to what is termed the piezophotovoltaic effect. This enhanced BPVE is highly sensitive to the crystallographic orientation induced by strain. In this strain-modified state, the photocurrent experiences a remarkable increase due to the asymmetric distribution of photo-excited carriers, unlocking new potential for optimizing photovoltaic performance in semiconductors through mechanical manipulation^2,7^. Despite this progress, the generated photocurrent remains in the nanoampere range, and numerous challenges in device engineering must be tackled to further advance these devices. One critical issue is the non-ohmic contact between metals and the photoactive transition metal dichalcogenide (TMD) layer, primarily due to the strong Fermi level pinning effect^8,9^. This problem complicates the precise evaluation of the BPVE, as the Schottky barrier formed at the interface substantially reduces BPVE-induced currents. This suppression remains largely unexplored, making it a significant hurdle in the development of more efficient BPVE-based devices.

Recent research published in Light: Science & Applications by Lain Jong Li’s research group from the University of Hong Kong presents an innovative design framework aimed at mitigating ohmic contact losses^10^. The study underscores the efficacy of employing bismuth (Bi) semimetal as an edge contact to overcome these critical challenges. In order to maximize contact characteristics, Bi was designed to firmly adhere to TMD layers and generate significant tensile strain. Greater photocurrent extraction is made possible by the edge contact’s lateral structure, which provides complete access to in-plane polarization from underlying TMD layers exposed to light. This is in contrast to the traditional top contact, which lacks interlayer transport (vertical transport). The fabrication involved depositing Bi/Au electrodes following CF_4_ plasma treatment of pre-patterned TMD regions and is as illustrated in Fig. 1. The edge contact device outperformed the top contact device, which had a photocurrent of 48.11 nA and a voltage of 1.65 mV, when irradiated with a linearly polarized laser (395 μW). There were notable improvements in photocurrent (1.26 μA) and voltage (39.44 mV), marking the highest photocurrent recorded for this class of material to date. Spatial distribution measurement studies indicate that edge contact devices exhibit a depinning effect with TMD layers, resulting in a reduced Schottky barrier and facilitating higher photocurrent extraction to the electrodes^11^. This BPVE originates from the strain naturally induced by the Bi electrode on the TMD structure, stemming from interface chemical bonding and a mismatch in thermal expansion coefficients^12^. The strain from the Bi electrode induces in-plane inversion symmetry breaking within the TMD layer, generating a strong polarization field and promoting lateral carrier transport across the TMD interlayers, leading to efficient carrier collection at the edge contact electrodes^5,13,14^. Additionally, applying external strain similar to the studies conducted by the Iwasa group further enhances the separation of electron-hole pairs within the TMD layers, promoting the overall photocurrent generation^15^. This configuration offers a significant advantage over conventional top contact systems due to the direct interaction with the lateral edges of the TMD structure. Furthermore, a purposeful design of a 3R-MoS_2_/WSe_2_ (BPVE/PVE) p-n heterojunction was made and examined for the first time. By properly developing heterojunctions based on BPVE material, BPVE appears to have considerable promise for enhancing solar cell performance and breaking the SQ limit.

**Fig. 1:**
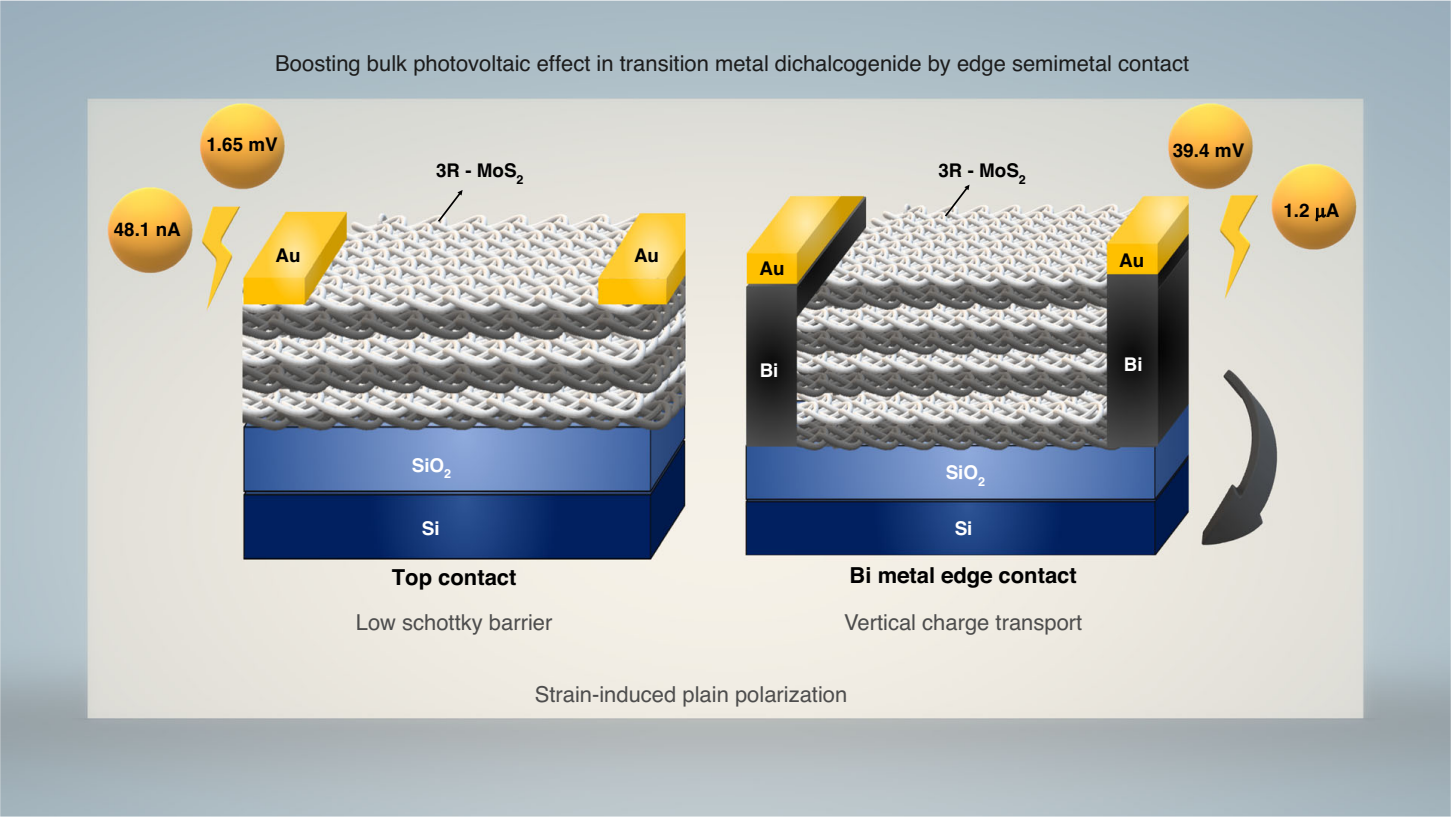
Schematic representation of conventional top contact and novel bi-metal edge contact of transition metal dichalcogenide solar cells

This highlights the intriguing potential of bismuth (Bi) semimetal as an edge contact for bulk photovoltaic systems based on TMDs. The high adhesion and induced tensile strain between Bi and TMD layers, enabled by interface chemical bonding and thermal expansion mismatch, resulted in a low Schottky barrier and increased carrier extraction efficiency. By leveraging in-plane polarization and overcoming the vertical transport limitations of top contact systems, the lateral configuration achieves notable improvements in both photocurrent and voltage. Additionally, the polarization field that results from the induced in-plane inversion symmetry breakdown enhances lateral carrier movement, supporting effective collection at the edge contacts and exhibiting tremendous photocarrier collection efficiency over conventional strategies. Through carefully designed heterostructures, BPVE-based materials can strategically overcome the Shockley–Queisser efficiency limit. BPVE and TMD research using edge contacts such as Bi semimetal will contribute to high-efficiency solar cells that surpass the SQ limit and offer innovative energy solutions. These materials might enable flexible, lightweight solar panels for wearables and portable devices. Better BPVE properties can be used for sensors, photodetectors, and optical modulators to increase the performance of optoelectronic devices. Bi-contact strain engineering presents opportunities for adaptable photonic systems and flexible electronics. Additionally, electronics strengthened by BPVE may be included in quantum and nanoscale energy applications and support self-powered technology.
